# Thermographic ranges of dromedary camels during physical exercise: applications for physical health/welfare monitoring and phenotypic selection

**DOI:** 10.3389/fvets.2023.1297412

**Published:** 2023-12-19

**Authors:** Carlos Iglesias Pastrana, Francisco Javier Navas González, Elena Ciani, Carmen Marín Navas, Juan Vicente Delgado Bermejo

**Affiliations:** ^1^Department of Genetics, Faculty of Veterinary Sciences, University of Cordoba, Cordoba, Spain; ^2^Department of Biosciences, Biotechnologies and Environment, University of Bari ‘Aldo Moro’, Bari, Italy

**Keywords:** thermo-physiological response, exercise tolerance, camel welfare, camel phenotyping, genetic selection

## Abstract

Despite the relatively wide knowledge of camel biomechanics, research into the immediate functional response that accompanies the execution of physical exercise remains unapproached. Therefore, selective breeding programs lack an empirical basis to achieve genetic improvement of physical stress tolerance traits and monitor camel welfare in this regard. Given the fact that physical exercise increases net heat production, infrared thermography (IRT) was selected to study the temperature changes at the skin surface of the different body areas in clinically normal dromedary camels, mostly relegated to leisure activities. Specifically, a lower dispersion at the individual level of the surface temperature at the scapular cartilage region, shoulder joint, and pelvis region, as well as lower values for Tmax and Tmin at the region of the ocular region, pectoral muscles, semimembranosus-semitendinosus muscles, and hind fetlock after exercise, have to be considered as breeding criteria for candidate selection. Such thermophysiological responses can be used as indirect measures of tissue activity in response to exercise and hence are reliable indicators of animal tolerance to physical exercise-induced stress. Additionally, sex, castration, age, and iris pigmentation significantly impacted the thermo-physiological response to exercise in the study sample, which can be attributed to hormones, general vigor, and visual acuity-mediated effects. These specific factors’ influence has to be considered for the evaluation of physical performance and the design of selection schemes for physical-related traits in dromedaries.

## 1 Introduction

The socioeconomic interest in camel breeding fundamentally resides in the production of food (milk and meat) and other products and by-products such as wool, dung, skin, and fat. Abundant literature does exist on the characterization of camel-based food products and subproducts, with special reference to the different animal-and management-related variables that potentially influence yield and thus economic benefits in these camel farming systems (1–5). However, several camel breeds are typically selected for their power and speed within a functional scenario of leisure, load-carrying, and traction work (6). Comparatively, little information is available for these specific tasks.

Elementary analyses of the biomechanics of gait (7–9), the elastic extension of tendons (10), the morphology of some parts of the distal skeleton (7, 11–13), and the pedal anatomy (14) in one-humped camels shed light on the normal movements, osteomuscular composition, and locomotor adaptations of these animals to sandy terrains in desert ecosystems. More specifically, the repercussions that different exercise conditions have at a biochemical and hormonal level are relatively widely studied (15–17), highlighting the near-Newtonian behavior of camel blood as a response to endurance activities (18). In regards to animal-dependent factors, Al-Shorepy (19) concludes that age and sex have a significant effect on racing performance.

Notwithstanding this conglomerate of empirical knowledge on camel biomechanics, research into the basic physiology and function of the musculature in the working camel still remains unapproached. The knowledge of the immediate functional responses and organic adaptations that accompany the execution of a session of physical activity or exercise allows the accurate, early diagnosis of decreased levels of sports performance and the design of training/rehabilitation programs (20, 21). Since physical exercise has a remarkable direct effect on net heat production (22), infrared thermography (IRT) is the technique of choice to study the underlying physiological response of muscles and other organic tissues during and after exercise (23). This non-invasive imaging method is capable of detecting temperature changes (thermogenesis) at the skin surface that are an indirect measure of tissue activity (local blood flow and metabolism rate) in response to exercise (24, 25).

In this study, using thermography, we examine 22 body regions to determine their significance in monitoring the effects of exercise in clinically normal dromedary camels. Additionally, we will analyze the varying impact of different factors specific to the animals on the thermo-physiological responses observed in these body regions. The results obtained will serve to evaluate the potential of infrared thermography to be used as a tool for reliable assessment of camel physical fitness and, thus, welfare impacts when these animals participate in physically demanding activities. The animal model used is the Canarian camel (*Camelus dromedarius*), an autochthonous endangered breed mainly relegated to leisure activities in which the physical health and performance of the animals have large significance (26).

## 2 Materials and methods

### 2.1 Animal sample and data selection

A total of 130 Canarian dromedaries (72 males and 58 females, aged between 18 months and 35 years old) were included in this study. All dromedary camels had resided at the respective participating farm for at least 1 year prior to the study. Animals were checked before the evaluations to ensure they were in good health.

### 2.2 Study duration

The study was carried out over 5 consecutive days in January 2021. For the study of the thermo-physiological response to exercise in leisure dromedaries, each dromedary camel participating in this study was exercised for 15 min by the same familiar handler. The exercise involved walking the animal for 15 min at a comfortable, active walking speed for each individual.

### 2.3 Thermal data collection

Thermal images for this study were captured using an Hti-Xintai HT-18 thermal imaging camera (220 × 160 IR resolution, 35,200 pixels). The camera has a thermal sensitivity of <0.05°C at temperatures of ≥30°C, a temperature detection range between −20°C and + 120°C, and an emissivity of 0.95.

To minimize the impact of environmental factors, thermographic evaluations were consistently conducted within an enclosed pen by the same technicians. Static thermal images were taken at a 90° angle from a distance of 1 m ± 50 cm from the animals, following recommendations from Yarnell et al. (23).

Thermal images were captured in 22 body regions. Thermal images were taken at three separate sampling time points: pre-exercise, post-exercise 0 min, and post-exercise 5 min.

### 2.4 Evaluated areas and thermal quantitative variables

The software used for thermographic assessment was Batch Flir Image Converter.^1^ Quantitative variables extracted from each thermal image included average temperature, maximum temperature, minimum temperature, and standard deviation, all of which were measured in degrees Celsius.

The evaluated areas from which the aforementioned quantitative variables were sampled comprised the ocular region (*Regio orbitalis*), scapular cartilage region (*Regio cartilaginis scapulae*), shoulder joint (*Articulatio humeri*), thorax (*Regio pectoris*), antebrachial region (*Regio antebrachii*), carpus region (*Regio carpi*), metacarpal region (*Regio metacarpi*), region of the proximal phalanx (*Regio phalangis proximalis*) of the thoracic limb, digital flexor tendons of the thoracic limb, coronary region of the thoracic limb (*Regio coronalis*), lateral and medial hoof cartilage of the thoracic limb (*Cartilago ungularis medialis et lateralis*), palmary bulb of the heel (*Torus ungulae*), lumbar region (*Regio lumbalis*), pelvis region (*Regio pelvis*), semimembranosus and semitendinosus muscles, tarsus region (*Regio tarsi*), metatarsus region (*Regio metatarsi*), region of the proximal phalanx (*Regio phalangis proximalis*) of the hind limb, digital flexor tendons of the hind limb, coronary region of the hind limb (*Regio coronalis*), the lateral and medial hoof cartilage of the hind limb (*Cartilago ungularis medialis et lateralis*), and plantary bulb of the heel (*Torus ungulae*). The “Nomina anatomica veterinaria” (Sixth Edition) (27) was used as a guideline to select the pertinent anatomical nomenclature used for the reference of the body regions considered in the present study.

The hump region was not evaluated due to its primarily fatty tissue composition, where fat thermogenesis primarily depends on the types of fatty acids present, which, in turn, are influenced by the animal’s diet and other factors related to the animal’s overall health status, factors that were not controlled in this study. Additionally, the femoro-patellar joint or stifle joint (*Articulatio genus*) was not assessed due to the presence of a notable hard callus (active osteoblasts that mineralize the callus matrix) with significant dimensions (area and thickness) in this region, which functions to protect this area when the camel sits on hot surfaces such as sand.

For the forelimb or thoracic limb (*Membrum thoracicum*) and hindlimb or pelvic limb (*Membrum pelvinum*), the same image was used to extract the average and standard deviation temperatures for each individual region evaluated within each limb. This approach provided a more precise evaluation of the workload and physiological stress relative to each individual region compared to the overall limb (forelimb or hindlimb).

### 2.5 Statistical analysis

In line with the methodologies outlined in González Ariza et al. (28), we initiated our evaluation by employing discriminant canonical analysis. This allowed us to create a tool that assesses optimal linear combinations of thermo-physiological response-related variables and animal-dependent factors able to determine within and between population clustering patterns across different moments of exercise [pre and post (0 and 5 min)].

Our independent variables comprised average temperature, maximum temperature, minimum temperature, and standard deviation per evaluated region. Additional variables such as sex, age (months), coat color (white, blonde, cinnamon, chestnut, bay, black, ashed, and roan), coat particularities (solid color, white-haired areas in extremities, white-haired areas in head and neck, white-haired areas in the thorax, white-haired areas in extremities, head and neck, white-haired areas in extremities and thorax, white-haired areas in head, neck and thorax, and white-haired areas across almost the body), eye color (brownish, brownish with blue spots, and bluish), neutering status (neutered and non-neutered), and active involvement (involved and not involved) in desensitization protocols were included.

Calculation of centroids for different moments of exercise [pre and post (0 and 5 min)] was conducted. The relative positions of each centroid were determined by substituting the mean values for the observations represented in each of the two detected discriminant functions (F1 and F2).

Subsequently, we computed squared Mahalanobis distances to measure dissimilarities between pre-exercise moments and post-exercise (0 and 5 min) moments. These squared Mahalanobis distances were determined using the following formula:


$$D_{ij}^{2}=\left({\bar{\gamma }}_{i}-{\bar{\gamma }}_{j}\right)\;{COV}^{-1}\left({\bar{\gamma }}_{i}-{\bar{\gamma }}_{j}\right),$$

where $D_{ij}^{2}$ is the distance between population i and j; ${\bar{\gamma }}_{i}$ and ${\bar{\gamma }}_{j}$ are the means of the variable x in the ith and jth populations, respectively; and COV^−1^ is the inverse of the covariance matrix of measured variable *x*. Finally, we used the squared Mahalanobis distance to visually illustrate the clustering patterns arising from variations in thermal information among the different exercise times [pre and post (0 and 5 min)] considered in this study. A dendrogram was then created using the underweighted pair-group method arithmetic averages (UPGMA) from Universität Rovira i Virgili (URV), Tarragona, Spain, and the phylogeny procedure of MEGA X 10.0.5 (Institute of Molecular Evolutionary Genetics, Pennsylvania State University, State College, PA, United States).

## 3 Results

**Figure 1:**
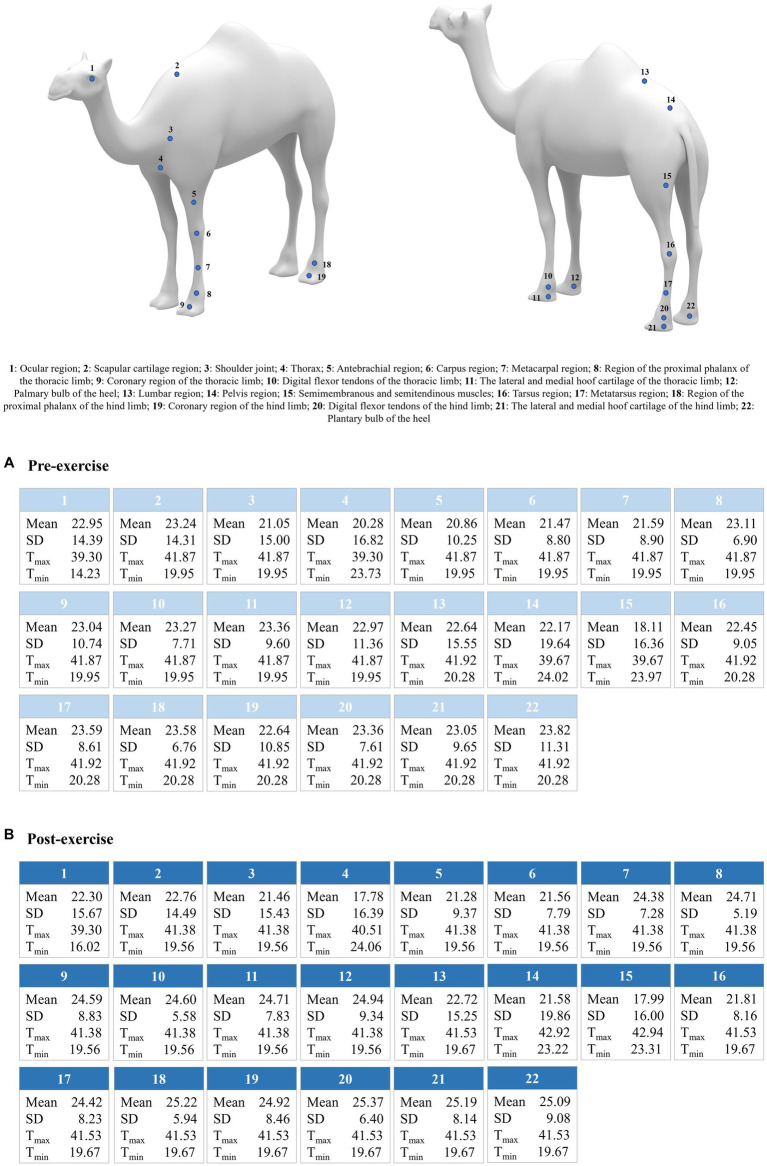
Average values for mean, standard deviation (SD), maximum (T_max_), and minimum (T_min_) temperature per body region and evaluation state (pre- and post-exercise 0 min). Data for post-exercise 5 min were not presented due to the lack of differences with pre-exercising, which suggests total thermal recovery of the animals after this time has passed.

A summary of the mean values for thermal information reported by all the variables considered across the body region sampled is shown in Figure 1.

Concerning the reliability of the discriminant canonical analysis model, after conducting 49 rounds of multicollinearity analyses, the variables included in the discriminant canonical analysis are shown in Table 1.

**Table 1:** Summary of the value of tolerance and VIF after multicollinearity analysis of thermal information-related variables in the Canarian camel breed.

Statistic	Tolerance (1−*R*^2^)	VIF (1/Tolerance)
Minimum temperature—Ocular region	0.6980	1.4326
Standard deviation of temperature—Metatarsus region	0.6819	1.4666
Standard deviation of temperature—Scapular cartilage region	0.6514	1.5353
Standard deviation of temperature—Carpus region	0.6406	1.5611
Standard deviation of temperature—Shoulder joint	0.6326	1.5808
Average temperature—Semimembranosus and semitendinous muscles	0.6234	1.6041
Standard deviation of temperature—Thorax	0.6149	1.6262
Standard deviation of temperature—Semimembranosus and semitendinous muscles	0.6059	1.6504
Standard deviation of temperature—Metacarpal region	0.6021	1.6609
Standard deviation of temperature—Ocular region	0.5994	1.6683
Age (months)	0.5947	1.6814
Average temperature—Thorax	0.5869	1.7040
Standard deviation of temperature—Lumbar region	0.5792	1.7265
Average temperature—Ocular region	0.5763	1.7354
Standard deviation of temperature—Tarsus region	0.5688	1.7580
Eye color—Brownish with blue spots	0.5650	1.7700
Eye color—Brownish	0.5507	1.8160
Standard deviation of temperature—Pelvis region	0.5464	1.8301
Standard deviation of temperature—Antebrachial region	0.5398	1.8524
Standard deviation of temperature—Region of the proximal phalanx of the hind limb	0.5164	1.9364
Standard deviation of temperature—Region of the proximal phalanx of the thoracic limb	0.5020	1.9920
Average temperature—Shoulder joint	0.4465	2.2395
Standard deviation of temperature—Palmary bulb of the heel	0.4199	2.3813
Minimum temperature—Region of the proximal phalanx of the hind limb	0.4156	2.4061
Average temperature—Lumbar regions	0.4100	2.4389
Average temperature—Scapular cartilage region	0.4068	2.4584
Standard deviation of temperature—Digital flexor tendons of the hind limb	0.3914	2.5548
Standard deviation of temperature—Plantary bulb of the heel	0.3687	2.7126
Standard deviation of temperature—Digital flexor tendons of the thoracic limb	0.3640	2.7474
Average temperature—Pelvis region	0.3600	2.7781
Minimum temperature—Thorax	0.3553	2.8149
Average temperature—Tarsus region	0.3539	2.8260
Average temperature—Antebrachial region	0.3374	2.9639
Standard deviation of temperature—The lateral and medial hoof cartilage of the thoracic limb	0.3244	3.0829
Standard deviation of temperature—Coronary region of the hind limb	0.3233	3.0927
Average temperature—Coronary region of the hind limb	0.3223	3.1026
Standard deviation of temperature—Coronary region of the thoracic limb	0.3086	3.2402
Minimum temperature—Semimembranosus and semitendinous muscles	0.2792	3.5811
Average temperature—Carpus region	0.2650	3.7735
Maximum temperature—Ocular region	0.2544	3.9306
Average temperature—Metatarsus region	0.2464	4.0586
Maximum temperature—Semimembranosus and semitendinous muscles	0.2436	4.1057
Sex—Male	0.2424	4.1246
Neutered—Yes	0.2313	4.3229
Standard deviation of temperature—The lateral and medial hoof cartilage of the hind limb	0.2225	4.4936
Average temperature—Metacarpal region	0.2181	4.5844
Maximum temperature—Thorax	0.2149	4.6534
Average temperature—Region of the proximal phalanx of the thoracic limb	0.2097	4.7691
Average temperature—Coronary region of the thoracic limb	0.2047	4.8861

Interpretation thumb rule: VIF ≥ 5 (highly correlated); 1 < VIF < 5 (moderately correlated); VIF > 1 (not correlated).

Pillai’s trace criterion reported a significant difference between pre- and post-exercise moments (Pillai’s trace criterion: 0.4434, F (observed value): 1.9764, F (critical value): 1.2695, df1: 98, df2: 680, *p*-value<0.0001), confirming the validity of the discriminant canonical analysis.

Out of the two functions identified through discriminant analysis, one was found to be significant for their discriminant ability (Table 2). Among these, the F1 function exhibited the highest discriminatory power, with an eigenvalue of 0.5374, explaining 83.84% of the variance.

**Table 2:** Canonical discriminant analysis efficiency parameters to determine the significance of each canonical discriminant function.

Test functions	Canonical correlations	Eigenvalue	Bartlett’s statistic	Discrimination (%)	*p*-value
1 through 2	0.5912	0.5374	191.8897	83.8425	0.0000
2	0.3063	0.1036	35.7705	16.1575	0.9038

The various variables examined in this study were ranked based on their discriminative capacity. An evaluation of the equality of group means of the dependent variables involved in the discriminant canonical analysis is presented in Table 3.

**Table 3:** Results for the tests of equality of group means to test for differences in the means across sample groups once redundant variables were removed.

Variable	Wilk’s Lambda	*F*	*p*-value	Rank
Standard deviation of temperature—Metacarpal region	0.9129	18.4583	<0.0001	1
Standard deviation of temperature—Region of the proximal phalanx of the thoracic limb	0.9173	17.4376	<0.0001	2
Standard deviation of temperature—Digital flexor tendons of the thoracic limb	0.9265	15.3445	<0.0001	3
Maximum temperature—Semimembranosus and semitendinous muscles	0.9273	15.1775	<0.0001	4
Standard deviation of temperature—The lateral and medial hoof cartilage of the thoracic limb	0.9549	9.1459	0.001	5
Standard deviation of temperature—Palmary bulb of the heel	0.9619	7.6602	0.001	6
Average temperature—Thorax	0.9630	7.4278	0.001	7
Average temperature—Coronary region of the hind limb	0.9649	7.0411	0.001	8
Standard deviation of temperature—Coronary region of the hind limb	0.9657	6.8670	0.001	9
Standard deviation of temperature—Plantary bulb of the heel	0.9689	6.2114	0.001	10
Standard deviation of temperature—Coronary region of the thoracic limb	0.9693	6.1206	0.001	11
Standard deviation of temperature—Carpus region	0.9744	5.0749	0.01	12
Standard deviation of temperature—Antebrachial region	0.9771	4.5267	0.01	13
Average temperature—Metacarpal region	0.9792	4.1033	0.02	14
Standard deviation of temperature—Ocular region	0.9822	3.5162	0.03	15
Maximum temperature—Ocular region	0.9839	3.1698	0.04	16
Standard deviation of temperature—Digital flexor tendons of the hind limb	0.9842	3.1018	0.05	17
Standard deviation of temperature—Tarsus region	0.9843	3.0787	0.05	18
Maximum temperature—Thorax	0.9849	2.9664	0.05	19
Average temperature—Coronary region of the thoracic limb	0.9851	2.9274	0.05	20
Standard deviation of temperature—Shoulder joint	0.9865	2.6566	0.0715	
Standard deviation of temperature—Pelvis region	0.9877	2.4128	0.0909	
Standard deviation of temperature—Thorax	0.9877	2.4110	0.0911	
Standard deviation of temperature—The lateral and medial hoof cartilage of the hind limb	0.9882	2.3194	0.0997	
Minimum temperature—Ocular region	0.9890	2.1567	0.1171	
Standard deviation of temperature—Region of the proximal phalanx of the hind limb	0.9896	2.0414	0.1312	
Standard deviation of temperature—Lumbar region	0.9911	1.7433	0.1763	
Average temperature—Region of the proximal phalanx of the thoracic limb	0.9920	1.5569	0.2121	
Average temperature—Shoulder joint	0.9934	1.2780	0.2798	
Average temperature—Metatarsus region	0.9940	1.1634	0.3135	
Average temperature—Carpus region	0.9951	0.9574	0.3848	
Average temperature—Pelvis region	0.9952	0.9322	0.3946	
Standard deviation of temperature—Metatarsus region	0.9955	0.8716	0.4191	
Minimum temperature—Semimembranosus and semitendinous muscles	0.9956	0.8524	0.4272	
Average temperature—Scapular cartilage region	0.9957	0.8292	0.4372	
Average temperature—Lumbar region	0.9961	0.7582	0.4692	
Average temperature—Tarsus region	0.9968	0.6136	0.5419	
Average temperature—Semimembranosus and semitendinous muscles	0.9981	0.3670	0.6930	
Standard deviation of temperature—Semimembranosus and semitendinous muscles	0.9983	0.3372	0.7140	
Average temperature—Ocular region	0.9984	0.3154	0.7297	
Standard deviation of temperature—Scapular cartilage region	0.9984	0.3121	0.7321	
Minimum temperature—Region of the proximal phalanx of the hind limb	0.9986	0.2762	0.7588	
Minimum temperature—Thorax	0.9992	0.1578	0.8541	
Average temperature—Antebrachial region	0.9993	0.1396	0.8697	
Age (months)	1.0000	0.0000	1.0000	
Sex—Male	1.0000	0.0000	1.0000	
Eye color—Brownish with blue spots	1.0000	0.0000	1.0000	
Eye color—Bluish	1.0000	0.0000	1.0000	
Neutered—Yes	1.0000	0.0000	1.0000	

Df1 = 2; Df2 = 387.

Higher values of F and lower values of Wilks’ lambda indicate greater discriminating power. The analysis revealed that standard deviation of temperature in the metacarpal region, standard deviation of temperature in the region of the proximal phalanx of the thoracic limb, standard deviation of temperature in the digital flexor tendons of the thoracic limb, maximum temperature in the semimembranosus and semitendinous muscles, standard deviation of temperature in the lateral and medial hoof cartilage of the thoracic limb, standard deviation of temperature in the palmary bulb of the heel, average temperature in the thorax, average temperature in the coronary region of the hind limb, standard deviation of temperature in the coronary region of the hind limb, standard deviation of temperature in the plantary bulb of the heel, standard deviation of temperature in the coronary region of the thoracic limb, standard deviation of temperature in the carpus region, standard deviation of temperature in the antebrachial region, average temperature in the metacarpal region, standard deviation of temperature in the ocular region, maximum temperature in the ocular region, standard deviation of temperature in the digital flexor tendons of the hind limb, standard deviation of temperature in the tarsus region, maximum temperature in the thorax, and average temperature in the coronary region of the thoracic limb made highly significant contributions (*p* < 0.0001) to the discriminant functions when qualitative gait evaluation levels were the clustering criteria.

Standardized discriminant coefficients were used to assess the relative weight of each dependent variable across the two established discriminant functions (Supplementary Table S1).

A Press’ Q-value of 51.80 (*n* = 390; *n*’ = 197; *K* = 3) was computed for thermal information across exercise moment levels [pre and post (0 and 5 min)], indicating that predictions can be considered better than chance at a 95% confidence level (29).

The results for the functions at the centroids are presented in Table 4.

**Table 4:** Functions at the centroids for the two discriminant functions detected in this study.

Function/Moment of exercise	Post-exercise 0 min	Post-exercise 5 min	Pre-exercise
1	−1.0064	0.7037	0.3027
2	0.1017	0.3318	−0.4334

**Figure 2:**
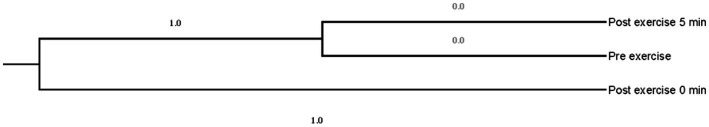
Dendrogram constructed from Mahalanobis distances across exercise moments [pre and post (0 and 5 min)].

Concerning the dendrogram constructed using the squared Mahalanobis distance (Figure 2), the absence of differences between pre- and post-exercise thermal information 5 min after exercise may be explained by the fact that camels are energy-efficient animals, hence it takes them less than 5 min to completely recover at a thermo-physiological level (14, 17, 30).

## 4 Discussion

The thermo-physiological response to physical exercise was evaluated through infrared thermography at 22 body regions of clinically normal dromedary camels. The mean temperature was generally higher after exercise in almost all the evaluated body regions, while the opposite trend was observed for the values of standard deviation, maximum temperature (T_max_), and minimum temperature (T_min_). As T_max_ and T_min_ do not decrease by approximately the same amount between evaluated time periods, the standard deviation is expected to decrease and the mean to move up, and vice versa (31). From a pure physiological viewpoint, the mean temperature of the body regions can be anticipated to be increased after physical exercise due to the near-immediate local rise in blood flow, nutrient supply, and metabolic rate, but also the temperature to be more or less homogeneous along each local body area depending on their specific functional repercussion in locomotion. In addition, considering the natural selection for energy efficiency of movement and optimization in extreme environments in camelids (32), a low-temperature dispersion should appear.

At the individual level, a more irregular regional temperature dispersion could be indicative of local functional alteration (e.g., microcirculation failures or inflammation) that might compromise animal physical health and thus performance (33). On the other hand, a higher regional temperature dispersion at the group level might be indicative of substantial interindividual variability for the thermo-physiological local response, which could be attributed to the effects of different animal-and environment-related factors and therefore plausible to be used as a reliable measure of individual tolerance to physical exercise-induced stress. This could also be reflecting the higher latency of thermo-physiological response signals to exercise at these body areas, which then indicates that thermographic evaluation of these areas should be prioritized to accurately examine tolerance to physical exercise.

This last particular thermo-physiological behavior is indeed patent for some of the examined body regions. For the ocular region, scapular cartilage region, pectoral muscles, pelvis region, and semimembranosus-semitendinosus muscles, the mean temperature is lower and the mean standard deviation and/or T_max_ and T_min_ are higher after exercise. Only at the shoulder joint area are the mean temperature and the mean standard deviation higher, and/or T_max_ and T_min_ are lower, after exercise. First, during exercise, as a potential stress-inducing activity, eyelid contractions and sensory inputs from the visual, vestibular, and somatosensory systems for the visual-motor coordination to maintain postural balance are enhanced (34, 35), which might be manifested in an increase in local temperature. Such visual-motor coordination, and thus the level of stress with which the animal has to deal, is in turn governed by different influencing factors related to the anatomy and general biomechanics of each individual. Moreover, such idiosyncrasy will be reflected in the variability of the thermo-physiological response at the scapular cartilage region, shoulder joint, and pelvis region, which are largely implicated in the attenuation of impact forces from the feet to the head and back during quadrupedal locomotion (36).

In regards to the generally higher mean maximum and minimum temperatures at the ocular region, pectoral muscles, pelvis region, and semimembranosus-semitendinosus muscles after exercise, the mean maximum temperature of the ocular region does not significantly vary between the resting state and after exercise, which could be attributed to the existence of a local critical temperature due to the greater risk of heat damage at the tissues of the eye and adjacent brain structures for their elevated sensitivity to the effects of heat (37). Furthermore, a higher mean T_max_ and/or T_min_ at the pectoral muscles, pelvis region, and semimembranosus-semitendinosus muscles after exercise could be explained by the potential role of such areas as external projection windows for cardiac work and blood flow at the level of the brachial plexus (38) and for the work of the caudal region of the musculature at hindlimb in the flexion-extension of both the hip or coxofemoral and stifle joints and their respective propulsive forces (23), respectively.

However, for the specific case of the tarsal region, the mean temperature, standard deviation, T_max_, and T_min_ are lower in the post-exercise state. Such a finding reveals the importance of the early physiological recovery of this area to maintain good mechanical performance (39), hence it can be used as a reliable indicator of physical health in these animal species. In the animal sample studied, as they are functionally relegated to riding activities, the mechanics of the hindlimbs are crucial for good performance and maintenance of good health status. This peculiar thermal behavior could also suggest the potential existence of an extraordinary blood vessel circulation involved in heat dissipation at this local body region, and that should be specifically approached in future applied studies.

In summary, the quantitative trends discussed, together with the discriminant potential between the resting and post-exercise states of the standard deviation values for surface temperature in practically all the regions evaluated, and to a lesser extent the mean temperature values and the mean edge values of the range, serve to construct a non-invasive protocol for the monitoring of the impact of physical exercise and welfare status in dromedary camels subjected to physical exercise. Such a statement is additionally reinforced with the percentages of correct classification of individuals based on discriminant analysis, from which a significant number of camels in resting state are classified by the discriminant analysis as if they would have performed physical exercise. Following the same logic, those camels that are classified as in a resting state after having performed exercise would be the individuals to be selected for breeding purposes since they are able to physiologically recover quickly after physical effort.

Specifically, the greater the variability in surface temperature values at the discriminating regions after exercise, the induced stress will presumably be greater, or, if it is the same, the individual tolerance to exercise and physical fitness will be lower. At a pragmatic level, such variability would be observed in the thermographic camera as a relatively heterogeneous map of colors and tones in the pertinent region(s). The T_max_ and the T_min_ at the region of the ocular region, pectoral muscles, semimembranosus-semitendinosus muscles, and the region of the proximal phalanx of the hind limb also have discriminant potential between the resting and post-exercise states’ surface temperatures. For the specific case of the ocular region, additional evidence is thus provided to the existing literature for other animal species, which reports that eye temperature is one of the most accurate, non-invasive tools to study animal welfare (40). For the thorax region, its discriminating potential reinforces the abovementioned significance of its role as a window of external projection for cardiac work and brachial plexus blood flow. Concerning the semimembranosus-semitendinosus muscles and the region of the proximal phalanx of the hind limb, these structures have a prominent implication in the coordination of movement and effectiveness of locomotion, mainly due to the control that they exert at the propulsive forces and their support of body weight in the animal model used (riding camels) (41). Although the T_min_ at the region of the proximal phalanx of the hind limb is the same as the other structures evaluated at the rear limbs for methodological reasons, its discriminating potential derives from the statistical relationships that exist between the thermo-physiological behavior at this body area and the pertinent for the remaining regions evaluated at the population level.

Then, the detection at these discriminating areas of thermo-physiological responses contrary to the indicated, specific trend or exceptional extreme values could be interpreted as the incidence of a functional alteration, probably subclinical, with local (e.g., osteomuscular alteration in hindlimbs) or systemic (e.g., cardiac pathology) repercussions. Notwithstanding, the determination of the possible deviation from the thermo-physiological behavior of a single individual and their preference to be included in the breeding schemes should be carefully implemented with consideration of the effects of other qualitative and quantitative factors such as sex, age, neutering status, and eye color. Males, neutered and young (sexually immature) animals, and camels with brownish and relatively spotted blue irises display greater variability in the thermo-physiological response to physical exercise. In contrast, ambient temperature, breed, and training level, but neither gender nor age, have significant effects on skin temperature in other sport animal species such as racehorses (42).

The impact of sex and neutering status on individual physiological recovery capacity after exercise in camels would be basically explained by hormonal mechanisms (e.g., androgen-mediated effects) (43) and the higher body corpulence after castration (44), as well as the differences between sexes for cell composition and skin structure in dromedaries (45). In relation to age, the lower general vigor and functional maturity of the thermoregulatory system in the early stages of development would determine a relative greater variability in the individual thermoregulatory abilities (46). Finally, the higher the heterogeneity in iris pigmentation, the lower the visual acuity may be (47), which may cause further stress when the visual effort to maintain proprioception during a physical exercise needs to be consistent.

Contrastingly, coat color particularities and training regimes did not significantly affect the body surface temperature in the study sample. The camel hair medulla, independent of their color and the possible influence of color on light reflection capacity, provides animals with a notable thermoregulatory capacity (48). These results are in accordance with those reported by Abdoun et al. (49), who found a non-significant effect of coat color on thermo-physiological responses and heat tolerance in Saudi dromedaries. Such an outcome would also explain why there are camels of very different colors, even dark ones, in desert-like environments. Finally, the non-significant influence of training regimens on thermo-physiological responses in camels could be derived from the above-cited physiological adaptations of these animals to energy optimization in arid and semi-arid environments.

Seeking routine, on-field application of the results derived from this research study, the thermographic ranges presented and discussed constitute a valuable tool for enhancing monitoring protocols to assess dromedary camel welfare before and after physically demanding activities. Camel breeders are provided with guidelines to refine their management practices and increase the list of selection criteria for genetic improvement and welfare-oriented purposes. Additionally, veterinarians will be able to detect, at early stages, subtle health alterations that are potentially limiting the physical performance of the leisure dromedary camels. Ultimately, incorporating thermographic technology into leisure-oriented activities not only promotes the ethical treatment of dromedary camels but also lays the foundation for a robust protocol that benefits both the animals and those responsible for their care. Furthermore, future applied thermography studies should focus on overcoming some current limitations, such as the measurement of rectal temperature and the analysis of biochemistry data, so that correlations between thermographic and physiological parameters can be established. By including age as a covariate in this information set, we could also estimate the life span of sports dromedaries. With this aim, when animals of different ages show appreciably higher surface temperatures and levels of stress-related biomarkers during the same physical activity, it can be deduced that their ability to tolerate physical exercise is lower. As a result, there is a greater likelihood of adverse effects on their overall wellbeing.

## 5 Conclusion

The lower the regional dispersion at the thermo-physiological response after physical exercise, the more likely it is to discard potential local functional alterations with a possible systemic impact. Moreover, greater individual tolerance to physical exercise-induced stress and, thus, a lesser negative impact on animal welfare can be assumed. Specifically, a lower dispersion of the surface temperature at the scapular cartilage region, shoulder joint, and pelvis region should be preferably used to select dromedary camels for traits related to tolerance to physical exercise-related stress, given that larger overall data variability was detected at these body regions within the study population. In addition, the edge values of the temperature range at the local level for the ocular region, thorax, semimembranosus-semitendinosus muscles, and region of the proximal phalanx of the hind limb have a discriminating effect in this regard, and camels with lower values for T_max_ and T_min_ at these areas after exercise have to be prioritized for selection with genetic improvement purposes. Sex, castration, age, and iris pigmentation also significantly affect the thermo-physiological response to exercise in dromedaries, which can be related to variations in stress levels because of hormones, general vigor, and visual acuity-mediated effects. The discussed thermographic ranges in this research study serve as a reliable, non-invasive tool for improving monitoring protocols for dromedary camel welfare, especially before and after physically demanding activities. The results offer guidelines for camel breeders to enhance their management practices and enable veterinarians to detect early signs of health alterations. Future applied studies are also encouraged to correlate thermographic measurements with other physiological data as well as estimate the life span of sports dromedaries by including age as a covariate in the combined monitoring (non-invasive and invasive techniques) of tolerance to physical exercise.

## Data availability statement

The original contributions presented in the study are included in the article/Supplementary material, further inquiries can be directed to the corresponding author.

## Ethics statement

All farms included in our study adhered to specific codes of good practices, ensuring humane care for the animals, in accordance with the national guidelines for the care and use of laboratory and farm animals in research. Written consent was obtained from the owners for their participation in the study. Our research was conducted in compliance with the Declaration of Helsinki. The Spanish Ministry of Economy and Competitiveness, as well as the Ethics Committee of Animal Experimentation at the University of Córdoba, authorized the application of the protocols used in this study. This authorization was granted in accordance with the 5th section of the 2nd article of the Royal Decree Law 53/2013, as the animals assessed were intended for accredited zootechnical purposes. This national decree adheres to the European Union Directive 2010/63/EU, issued on September 22, 2010.

## Author contributions

CP: Data curation, Formal analysis, Investigation, Software, Visualization, Writing – original draft, Writing – review & editing. FN: Conceptualization, Data curation, Formal analysis, Investigation, Methodology, Resources, Software, Supervision, Validation, Visualization, Writing – original draft, Writing – review & editing. EC: Conceptualization, Funding acquisition, Project administration, Resources, Supervision, Validation, Visualization, Writing – review & editing. CM: Data curation, Formal analysis, Investigation, Methodology, Software, Writing – review & editing. JD: Funding acquisition, Project administration, Resources, Supervision, Validation, Visualization, Writing – review & editing.

## Footnotes

^1^ http://test.daves.cz/wrapper/wrapper.zip
