# Supplementary material for: Thermographic ranges of dromedary camels during physical exercise: applications for physical health/welfare monitoring and phenotypic selection
Source: Front Vet Sci. 2023 Dec 19;10:1297412. doi: 10.3389/fvets.2023.1297412 (PMC10762792; doi:10.3389/fvets.2023.1297412)
Supplement: Supplementary file 1 [file Table_1.docx]

**Supplementary Table S1.** Standardized canonical discriminant function coefficients.

| **Dependent variable** | **F1** | **F2** |
| --- | --- | --- |
| Age (months) | -0.0143 | 0.0810 |
| Minimum Temperature-Ocular region | -0.0073 | 0.2939 |
| Maximum Temperature-Ocular region | 0.4359 | -0.4010 |
| Average Temperature-Ocular region | 0.0828 | -0.1102 |
| Standard Deviation of Temperature-Ocular region | -0.1210 | 0.4096 |
| Average Temperature-Shoulder joint | 0.0983 | -0.1354 |
| Standard Deviation of Temperature-Shoulder joint | -0.0338 | 0.1848 |
| Minimum Temperature-Thorax | -0.1863 | -0.2256 |
| Maximum Temperature-Thorax | -0.0185 | 0.5843 |
| Average Temperature-Thorax | 0.1149 | -0.1962 |
| Standard Deviation of Temperature-Thorax | 0.1351 | 0.0019 |
| Average Temperature-Antebrachial region | -0.1090 | -0.0871 |
| Standard Deviation of Temperature-Antebrachial region | 0.0014 | -0.0653 |
| Average Temperature-Lumbar region | -0.1062 | -0.0233 |
| Standard Deviation of Temperature-Lumbar region | 0.1003 | 0.2180 |
| Average Temperature-Pelvis region | 0.4429 | -0.1373 |
| Standard Deviation of Temperature-Pelvis region | -0.2332 | -0.0728 |
| Average Temperature-Scapular cartilage region | 0.3177 | -0.2614 |
| Standard Deviation of Temperature-Scapular cartilage region | -0.2071 | 0.0665 |
| Minimum Temperature- Semimembranous and semitendinous muscles | 0.3574 | 0.2380 |
| Maximum Temperature-Semimembranous and semitendinous muscles | -0.7499 | 0.0576 |
| Average Temperature- Semimembranous and semitendinous muscles | -0.1604 | -0.1120 |
| Standard Deviation of Temperature- Semimembranous and semitendinous muscles | -0.0063 | -0.0655 |
| Average Temperature-Carpus region | 0.3765 | 0.0188 |
| Standard Deviation of Temperature-Carpus region | 0.0658 | 0.0724 |
| Average Temperature-Metacarpal region | -0.3745 | 0.6742 |
| Standard Deviation of Temperature-Metacarpal region | 0.4042 | 0.2232 |
| Average Temperature-Region of the proximal phalanx of the thoracic limb | -0.1447 | 0.1903 |
| Standard Deviation of Temperature-Region of the proximal phalanx of the thoracic limb | 0.1915 | 0.2059 |
| Standard Deviation of Temperature-Digital flexor tendons of the thoracic limb | 0.1898 | -0.0463 |
| Average Temperature-Coronary region of the thoracic limb | -0.0594 | -0.4102 |
| Standard Deviation of Temperature-Coronary region of the thoracic limb | -0.0380 | -0.2009 |
| Standard Deviation of Temperature-The lateral and medial hoof cartilage of the thoracic limb | 0.1276 | 0.3083 |
| Standard Deviation of Temperature-Palmary bulb of the heel | 0.1322 | -0.3596 |
| Average Temperature-Tarsus region | 0.0048 | 0.1440 |
| Standard Deviation of Temperature-Tarsus region | 0.0871 | 0.0967 |
| Average Temperature-Metatarsus region | 0.2536 | -0.4176 |
| Standard Deviation of Temperature-Metatarsus region | -0.0333 | -0.0179 |
| Minimum Temperature-Region of the proximal phalanx of the hind limb | 0.0123 | -0.1790 |
| Standard Deviation of Temperature-Region of the proximal phalanx of the hind limb | -0.0495 | 0.0099 |
| Standard Deviation of Temperature-Digital flexor tendons of the hind limb | 0.0395 | 0.1819 |
| Average Temperature-Coronary region of the hind limb | -0.3510 | 0.0976 |
| Standard Deviation of Temperature-Coronary region of the hind limb | 0.3427 | -0.0178 |
| Standard Deviation of Temperature-The lateral and medial hoof cartilage of the hind limb | -0.4245 | -0.2261 |
| Standard Deviation of Temperature- Plantary bulb of the heel | 0.2410 | -0.1001 |
| Sex-Male | 0.0627 | 0.0865 |
| Sex-Female | 0.0000 | 0.0000 |
| Eye colour-Brownish | 0.0000 | 0.0000 |
| Eye colour-Brownish with blue spots | -0.1028 | -0.0381 |
| Eye color-Bluish | 0.1451 | -0.0218 |
| Neutered-Yes | 0.0407 | 0.0407 |
| Neutered-No | 0.0000 | 0.0000 |
